# Regulation of the *Drosophila Enhancer of split* and *invected-engrailed* Gene Complexes by Sister Chromatid Cohesion Proteins

**DOI:** 10.1371/journal.pone.0006202

**Published:** 2009-07-09

**Authors:** Cheri A. Schaaf, Ziva Misulovin, Gurmukh Sahota, Akbar M. Siddiqui, Yuri B. Schwartz, Tatyana G. Kahn, Vincenzo Pirrotta, Maria Gause, Dale Dorsett

**Affiliations:** 1 Edward A. Doisy Department of Biochemistry and Molecular Biology, Saint Louis University School of Medicine, Saint Louis, Missouri, United States of America; 2 Department of Genetics, Washington University School of Medicine, Saint Louis, Missouri, United States of America; 3 Microarray Core Facility, Molecular Microbiology and Immunology, Saint Louis University School of Medicine, Saint Louis, Missouri, United States of America; 4 Department of Molecular Biology and Biochemistry, Rutgers University, Piscataway, New Jersey, United States of America; Texas A&M University, United States of America

## Abstract

The cohesin protein complex was first recognized for holding sister chromatids together and ensuring proper chromosome segregation. Cohesin also regulates gene expression, but the mechanisms are unknown. Cohesin associates preferentially with active genes, and is generally absent from regions in which histone H3 is methylated by the Enhancer of zeste [E(z)] Polycomb group silencing protein. Here we show that transcription is hypersensitive to cohesin levels in two exceptional cases where cohesin and the E(z)-mediated histone methylation simultaneously coat the entire *Enhancer of split* and *invected-engrailed* gene complexes in cells derived from *Drosophila* central nervous system. These gene complexes are modestly transcribed, and produce seven of the twelve transcripts that increase the most with cohesin knockdown genome-wide. Cohesin mutations alter eye development in the same manner as increased *Enhancer of split* activity, suggesting that similar regulation occurs in vivo. We propose that cohesin helps restrain transcription of these gene complexes, and that deregulation of similarly cohesin-hypersensitive genes may underlie developmental deficits in Cornelia de Lange syndrome.

## Introduction

The cohesin protein complex holds sister chromatids together, ensuring their proper segregation upon cell division [1]–[3]. Cohesin has a ring-like structure that encircles DNA [4], [5], formed by the Smc1, Smc3, Rad21 and Stromalin (SA) proteins. In most organisms, cohesin binds chromosomes throughout interphase, and several findings indicate that it regulates gene expression. The *Drosophila* Nipped-B protein that loads cohesin onto chromosomes facilitates activation of the *cut* and *Ultrabithorax* homeobox genes, and cohesin inhibits *cut* expression [6]–[9]. *Drosophila* cohesin facilitates expression of a steroid hormone receptor and axon pruning in non-dividing neurons [10], [11], and the Rad21 cohesin subunit encoded by *verthandi* (*vtd*), was identified genetically by its opposing effect to Polycomb group (PcG) silencing of homeotic genes [12], [13]. Rad21 also facilitates expression of zebrafish Runx genes in a cell-type specific manner [14].

To understand how Nipped-B and cohesin regulate gene expression, their binding was mapped in the genomes of *Drosophila* cultured cells, revealing that they co-localize genome-wide [15]. Cohesin was also mapped in the human genome [16], and in 3% of the mouse genome [17]. All three studies show that cohesin binds many genes, and that binding is particularly enriched around transcription start sites.

In mammals, cohesin co-localizes extensively with the CCCTC-binding factor (CTCF) that functions as a transcriptional insulator, and cohesin contributes to insulation [16], [17]. CTCF is thought to function by forming long-range chromosome loops, and cohesin and CTCF support transcription-dependent loops in the human apoliporotein gene cluster [18] and a developmentally-regulated loop at the *IFNG* cytokine locus in mammalian T cells [19].

There are also links between insulators and cohesin in *Drosophila*. A 75 kb domain of cohesin that covers the active *Abd-B* gene in the bithorax complex is flanked by a CTCF site near the 5′ end of *Abd-B*, and the Fab-7 insulator downstream of *Abd-B* [15], [20], suggesting that insulators define some cohesin domains. On the basis of genetic evidence it was suggested that cohesin blocks enhancer-promoter interactions in *cut*, and that Nipped-B counters this insulation by controlling cohesin binding [8]. Most recently, genome-wide mapping revealed that the *Drosophila* CP190 insulator protein co-localizes extensively with cohesin [21].

Many differences in cohesin binding between different *Drosophila* cell lines correlate with differences in transcription, with cohesin binding a gene only in those cells in which the gene is active [15]. Cohesin extensively overlaps RNA polymerase II (PolII) genome-wide, but is almost always absent from regions in which the E(z) protein of the PRC2 PcG silencing protein methylates histone H3 on the lysine 27 residue (H3K27Me3).

There are rare cases where cohesin overlaps H3K27Me3 over large regions in ML-DmBG3 (BG3) cells [22] derived from *Drosophila* central nervous system. One of these is the *Enhancer of split* complex [E(spl)-C] that contains twelve genes, including seven basic helix-loop-helix (bHLH) genes that repress neural fate [23]. Another is the *invected-engrailed* complex with two homeobox genes expressed in posterior developmental compartments [24]–[26]. The unusual pattern prompted us to determine if cohesin regulates these gene complexes. We find that genes in these complexes are expressed at modest levels, and that in sharp contrast to most cohesin-binding genes, reducing Nipped-B or cohesin levels dramatically increases their transcription.

## Results

Cohesin and RNA polymerase II (PolII) binding overlap extensively genome-wide, while cohesin shows a negative correlation with the H3K27Me3 mark made by the PRC2 PcG silencing complex [15]. PcG target genes such as *Abd-B* or *cut* bind little or no cohesin in cells in which they are silenced, but bind cohesin over large regions of 75 and 150 kb in cells in which they are transcribed [15].

**Figure 1:**
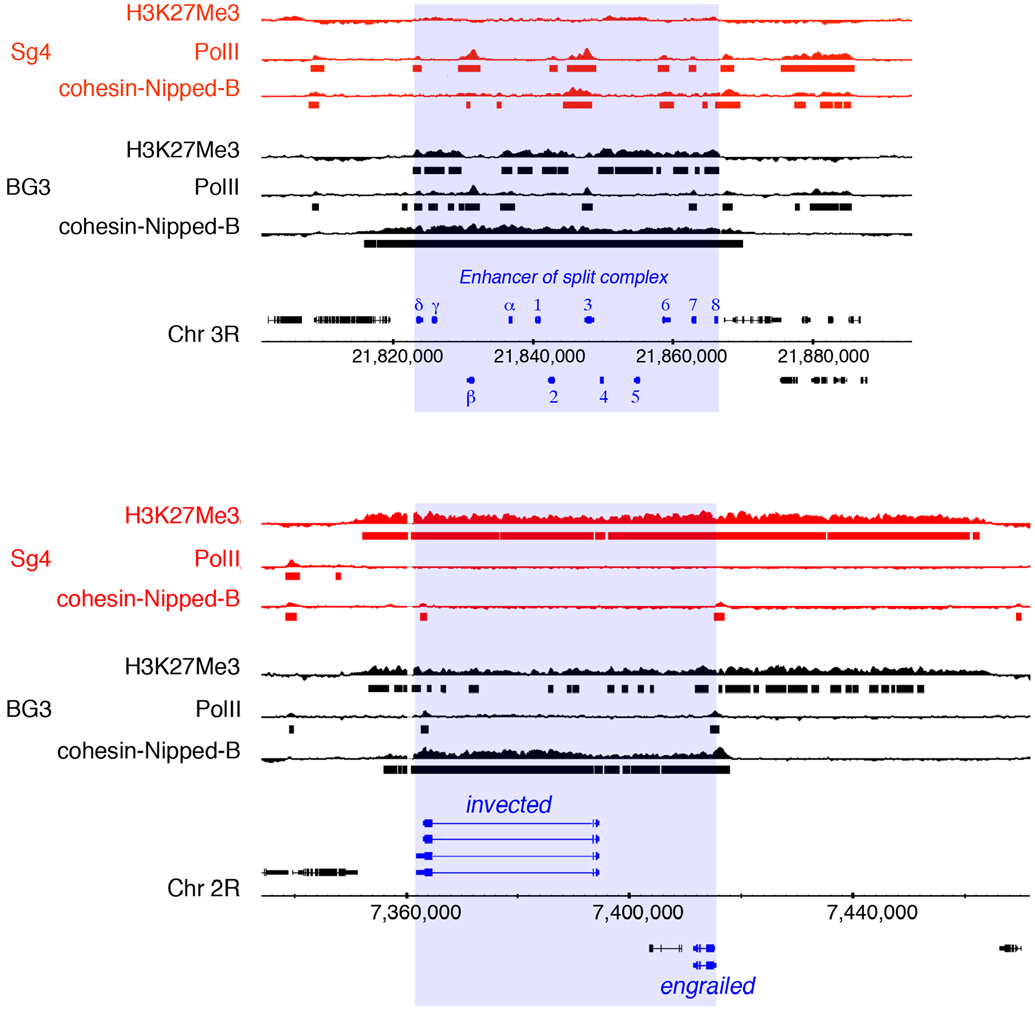
*Enhancer of split* and *invected-engrailed* gene complexes. The *Enhancer of split* complex [E(spl)-C] (top) contains twelve genes (blue): *HLHmδ*, *HLHmγ*, *HLHmβ*, *mα*, *m1*, *m2*, *HLHm3*, *m4*, *HLHm5*, *m6*, *HLHm7*, and *E(spl)m8*. Nucleotide numbering is from the April 2006 genome (Berkeley Drosophila Genome Project). Genes above the scale are transcribed from left to right, and those below from right to left. Tracks above the gene diagrams show chromatin immunoprecipitation data for histone H3 lysine 27 trimethylation (H3K27Me3), RNA polymerase II (PolII) and combined cohesin and Nipped-B binding (cohesin-Nipped-B) for Sg4 (red) and BG3 cells (black) [15,56, Y.B. Schwartz, T.G. Kahn, P. Stenberg, K. Ohno, R. Bourgon, and V. Pirrotta, submitted). Bars below each track show regions that bind at p≤10^−3^, as determined using the MAT program. The bottom shows the same for the *invected-engrailed* complex.

While comparing the cohesin and H3K27Me3 patterns, we noted eight unusual regions of extensive overlap ranging in length from 4.8 to 80.9 kb in the genome of BG3 cells derived from central nervous system, and only two such regions in Sg4 cells of embryonic origin (Table S1). Strikingly, two of the BG3-specific overlaps align perfectly with developmentally-important gene complexes. Figure 1 shows the association of cohesin, RNA polymerase II (PolII), and H3K27Me3 with the *Enhancer of split* and *invected-engrailed* complexes in BG3 and Sg4 cells. In BG3 cells, the 50 kb length of the E(spl)-C binds cohesin and has extended regions of H3K27Me3. Six genes (*HLHmδ*, *HLHmβ*, *mα*, *HLHm3*, *HLHm7*) bind PolII. By contrast, in Sg4 cells, only three E(spl)-C genes bind cohesin (*HLHmβ*, *HLHm3*, *m6*), six bind PolII (*HLHmδ*, *HLHmβ*, *m2*, *HLHm3*, *m6*, *HLHm7*), and there is no H3K27Me3. Similar to the E(spl)-C, the *invected-engrailed* complex is also coated by cohesin, and has extensive H3K27Me3 in BG3 cells (Figure 1). The cohesin domain extends from upstream of the *invected* transcription start site to a region upstream of *engrailed* that contains a Polycomb Response Element (PRE) and sequences required for interactions with transcriptional enhancers [27]. The H3K27Me3 region also starts upstream of *invected*, but extends 50 kb past the PRE, over a region that regulates *engrailed* [28]. In Sg4 cells, H3K27Me3 also coats the *invected-engrailed* complex and the regulatory region, but there is no PolII and little cohesin, as is typical for PcG-targeted genes [15].

### Cohesin Regulates the E(spl)-C and *invected-engrailed* Complex in BG3 Cells

**Figure 2:**
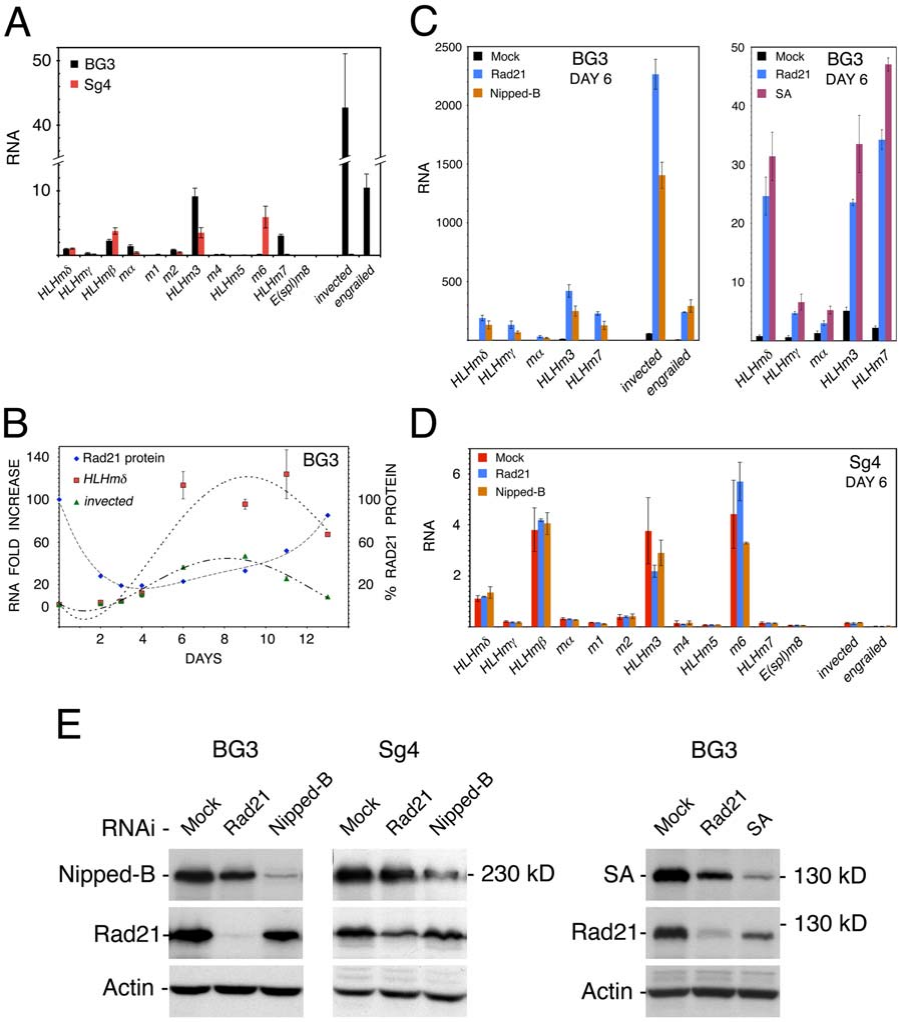
Regulation of the E(spl)-C and *invected-engrailed* complex by cohesin and Nipped-B. (A) Transcripts for the E(spl)-C and *invected-engrailed* complex in BG3 (black) and Sg4 (red) cells quantified by RT-PCR and normalized to *RpL32*. The *HLHmδ* level in BG3 cells is defined as 1 unit, and all transcripts are normalized to this value. By comparison to genomic DNA standards, *HLHmδ* transcripts in BG3 cells are 8,400-fold less than *RpL32* transcripts. BG3 values are the average of three RNA preparations, and Sg4 values are the average of two. Standard errors were calculated using all RT-PCR replicates from all biological replicates. (B) Rad21 RNAi time course, for Rad21 protein (blue diamonds, 100% starting), and fold-increases for the *HLHmδ* (red squares) and *invected* (green triangles) transcripts. Similar time courses are seen for *engrailed* and other E(spl)-C transcripts (not shown). Nipped-B knockdown shows similar time courses in Nipped-B protein and E(spl)-C and *invected-engrailed* transcripts (not shown), except that some E(spl)-C transcripts decrease on day 3 (Figure 3). (C) The left panel shows transcript levels in a typical experiment with mock RNAi-treated BG3 cells (black) and BG3 cells six days after Rad21 (blue) or Nipped-B (orange) RNAi treatment. The right panel shows transcript levels in another experiment with mock-treated BG3 cells (black), and BG3 cells treated with Rad21 (blue) or SA (purple) RNAi six days after treatment. (D) E(spl)-C and *invected-engrailed* transcript levels in mock-RNAi treated Sg4 cells (red), or Sg4 cells after two successive 3 day Rad21 (blue) or Nipped-B (orange) RNAi treatments. (E) Western blots of whole cell extracts after RNAi treatment. The three left panels show the same blot of BG3 extracts six days after RNAi probed with Nipped-B, Rad21 and Actin antisera. RNAi treatments are indicated at the tops of the lanes. The middle three panels show a blot of Sg4 extracts after two successive 3 day RNAi treatments. The right panels show a blot of BG3 extracts probed with SA, Rad21 and Actin antibodies six days after RNAi.

The unusual cell-type specific overlap of cohesin and H3K27Me3 that covers the E(spl)-C and *invected-engrailed* raised the possibility that cohesin might regulate their expression. Genome-wide, 480 genes have H3K27Me3 (p≤10^−3^) in their transcribed regions in BG3 cells, and only 64 (13%) of these bind PolII, including the genes in the E(spl)-C and the *invected-engrailed* complex. Although PcG proteins bind PREs of some target genes in both the inactive and active states, for the genes examined, H3K27Me3 covers the transcribed region only when they are silent [29]–[32]. We measured transcripts to compare expression of the E(spl)-C and *invected-engrailed* complex in BG3 and Sg4 cells. Consistent with the binding of PolII, seven E(spl)-C genes (*HLHmδ*, *HLHmγ*, *HLHmβ*, *mα*, *m2*, *HLHm3*, *HLHm7*), *invected*, and *engrailed* are transcribed in BG3 cells (Figure 2A). An overlapping set of six E(spl)-C genes (*HLHmδ*, *HLHmβ*, *mα*, *m2*, *HLHm3*, *m6*) are expressed in Sg4 cells at levels similar to those seen in BG3 cells (Figure 2A), but *invected* and *engrailed* are essentially silent. Thus at the *invected-engrailed* complex, which is coated by H3K27Me3 in both cell types, the presence of Nipped-B and cohesin correlates with expression, suggesting that cohesin prevents complete silencing, and/or that incomplete silencing promotes cohesin binding.

We used RNAi to knock down Nipped-B and cohesin to see if this alters expression of the *Enhancer of split* and *invected-engrailed* complexes. Knockdown of Nipped-B had little effect on cohesin levels, while Rad21 knockdown slightly reduced SA as previously noted [33], and SA RNAi reduced Rad21 (Figure 2E). SA and Rad21 interact, making it likely that they stabilize each other. In several experiments with BG3 cells, knockdown of Nipped-B, Rad21 or SA was maximal within two days, and on the order of 80% for several days (Figure 2B,E). Knockdown in Sg4 cells was maximally 60% after two successive treatments.

We saw large increases in E(spl)-C, *invected* and *engrailed* transcripts in BG3 cells six days after Rad21, Nipped-B or SA RNAi in all of several experiments (Figure 2B,C). The increases varied somewhat between experiments. In Figure 2C, the *HLHmδ* transcripts increase 130-fold by day 6 in one experiment, and 25-fold in another with Rad21 RNAi, representing some of the largest and smallest increases observed in the nearly forty independent Rad21 RNAi experiments that were performed. Within each experiment using the same cell passage, however, effects were similar between Rad21 and Nipped-B knockdown, or between Rad21 and SA RNAi (Figure 2C). Thus we attribute the variability in the fold-effects from experiment to experiment to unknown differences in the physiology or growth state of the cells between passages, and conclude that overall, Nipped-B and cohesin have similar effects on gene expression. We measured transcripts up to 13 days after RNAi, when Nipped-B (not shown) or Rad21 (Figure 2B) recover. The E(spl)-C and *invected*-*engrailed* transcripts start to decrease, but are still above initial levels (Figure 2B).

Nipped-B or cohesin RNAi had little effect on expression of the E(spl)-C in Sg4 cells (Figure 2D), including the cohesin-binding *HLHm3* and *m6* genes. There was also no effect on the silenced *invected* and *engrailed* genes. Although Rad21 and Nipped-B knockdown was less efficient in Sg4 cells (Figure 2E), as shown below, Rad21 knockdown of 30 to 50% in BG3 cells alters E(spl)-C RNA levels. We conclude that the E(spl)-C and *invected-engrailed* are less sensitive to cohesin dosage in Sg4 than in BG3 cells, as might be expected from the substantial differences in cohesin binding between the two cell types.

**Figure 3:**
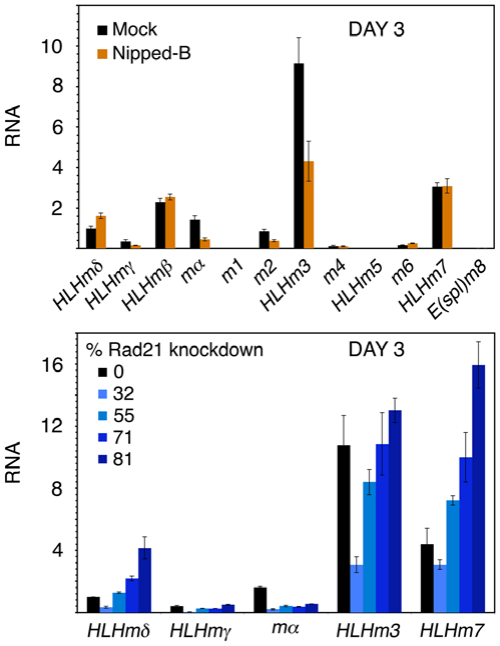
Biphasic changes in E(spl)-C transcripts after Nipped-B and Rad21 knockdown in BG3 cells. The top panel shows E(spl)-C transcript levels in mock-treated (black) or Nipped-B RNAi treated (orange) BG3 cells three days after treatment. Similar results were obtained in all Nipped-B RNAi experiments. All levels are relative to *HLHmδ* in mock-treated cells. The data shown is an average of two RNAi experiments. The bottom panel shows the indicated E(spl)-C transcript levels three days after treatments with increasing amounts of Rad21 dsRNA that cause different extents of knockdown (mock, 0%; 0.7 µg per 3 cm well, 32%; 1.7 µg, 55%; 3.3 µg, 71%; 6.7 µg, 81%).

On day 3 after Nipped-B RNAi, some E(spl)-C transcripts (*HLHmγ*, *mα*, *m2*, *HLHm3*) decrease (Figure 3), yet show large increases by day 6 (Figure 2). Similar decreases at day 3 were seen in all Nipped-B RNAi experiments. To see if a biphasic effect also occurs with Rad21, we used different amounts of dsRNA to control RNAi efficiency. A 30% knockdown decreased most E(spl)-C transcripts, while a 55% reduction decreased some and increased others (Figure 3). Thus Rad21 has a biphasic effect similar to Nipped-B.

E(spl)-C transcripts are miRNA targets [34], and we considered the possibility that cohesin knockdown decreases miRNA activity to increase transcript stability in BG3 cells. Rad21 knockdown, however, had little effect on the stability of E(spl)-C transcripts (Table S2), and we therefore conclude that cohesin RNAi elevates E(spl)-C transcription.

Nipped-B or Rad21 knockdown slowed but did not arrest cell division in BG3 or Sg4 cells, consistent with previous findings in *Drosophila* cells [33]. Sister chromatid separation increased 2 to 3-fold over controls, but there was no increase in hyperploid cells, indicating that the minor cohesion deficits did not affect segregation (Table S3). Nipped-B or cohesin RNAi did not increase cell death, as determined by trypan blue staining.

### Polycomb Represses the E(spl)-C in BG3 Cells

**Figure 4:**
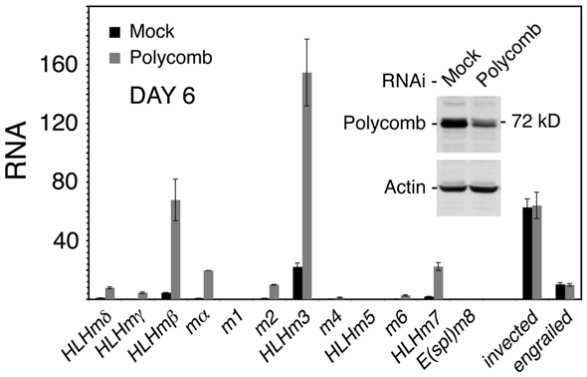
Effects of Polycomb on E(spl)-C and *invected-engrailed* transcripts in BG3 cells. The graph shows transcript levels in mock-treated BG3 cells (black) and in Polycomb RNAi-treated cells (gray) six days after treatment. The western blot shows the Polycomb protein knockdown (∼70%) on day 6. All transcripts are relative to *HLHmδ* in mock control cells. Similar results were obtained in three experiments.

In contrast to *engrailed*, the E(spl)-C has not previously been reported to be a PcG target. We used RNAi knockdown of the Polycomb (Pc) subunit of the PRC1 complex to see if PcG proteins repress the E(spl)-C in BG3 cells. With a Pc knockdown of some 70%, most E(spl)-C transcripts increased several-fold by day 6, indicating that in addition to cohesin, PRC1 restrains their expression (Figure 4). The *invected* and *engrailed* RNA levels did not change (Figure 4), although *Abd-B*, which is PcG-silenced and does not bind cohesin [15], showed up to 1200-fold increases in transcript levels with Pc knockdown (not shown). The lack of effects on *invected* and *engrailed* transcripts suggests that Pc is not strongly limiting for their repression in BG3 cells. Pc is only weakly limiting for repression of *engrailed* in embryos, and is less limiting than other PcG proteins for repression of many target genes in imaginal discs [31], [35].

### The CP190 Insulator Protein Weakly Regulates E(spl)-C and *invected-engrailed* Transcription in BG3 Cells

**Figure 5:**
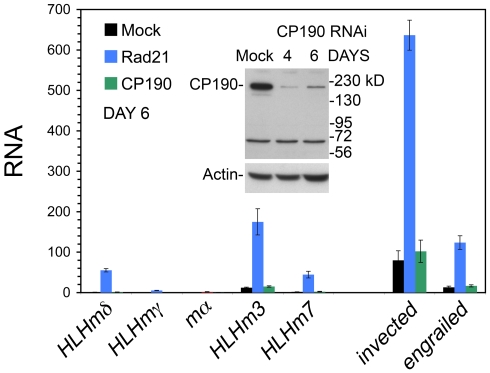
Effects of the CP190 insulator protein on E(spl)-C and *invected-engrailed* transcripts in BG3 cells. The graph shows transcript levels in mock-treated BG3 cells (black), Rad21 (blue) and CP190 (green) RNAi-treated BG3 cells six days after treatment. The western blot shows the knockdown of CP190 protein on days 4 and 6 (∼90%). The unlabeled protein under 72 kD in size that is unaffected by RNAi is a cross-reacting cytoplasmic protein (Marek Bartkuhn and Rainer Renkawitz, personal communication). Similar results were obtained with 4 and 8 days after CP190 RNAi.

In mammalian cells, cohesin can regulate gene expression by contributing to activity of the CTCF insulator protein and insulator-mediated looping [16]–[19]. Drosophila has many insulator proteins, including CTCF, Su(Hw), GAF, and BEAF. All co-localize extensively genome-wide with the CP190 protein, which is required for CTCF and Su(Hw) function, and likely also for GAF and BEAF insulator activities [21], [36]. CP190 also co-localizes extensively with cohesin on chromosomes [21]. We used RNAi to knockdown CP190 protein by some 90%, but in contrast to the 10 to 80-fold increases seen with Rad21 RNAi, there were maximally 1.2 to 2-fold increases in E(spl)-C and *invected-engrailed* transcripts six days after RNAi treatment (Figure 5). By day 8, Rad21 knockdown increased E(spl)-C and *invected-engrailed* transcripts 30 to 220-fold, but CP190 knockdown increased the transcripts only 1.7 to 3.4-fold (not shown). Thus the major role of cohesin in regulating these gene complexes is unlikely to be support of CP190 insulator activity.

### *Nipped-B* and *Rad21* Mutations Alter *Notch^split^* Mutant Phenotypes

We used mutant phenotypes of the *split* missense mutation in the *Notch* receptor gene (*N* ^spl-1^) that are sensitive to E(spl)-C activity to test if cohesin regulates the E(spl)-C in vivo. *N^spl-1^* reduces activation of proneural genes, thereby decreasing the number of photoreceptors in the eye, and altering bristles [37]. E(spl)-C duplications, the *E(spl)^D^* gain-of-function allele, and forced overexpression of some E(spl)-C genes increase the severity of the eye phenotype [37]–[40], while E(spl)-C deletions suppress [41].

**Figure 6:**
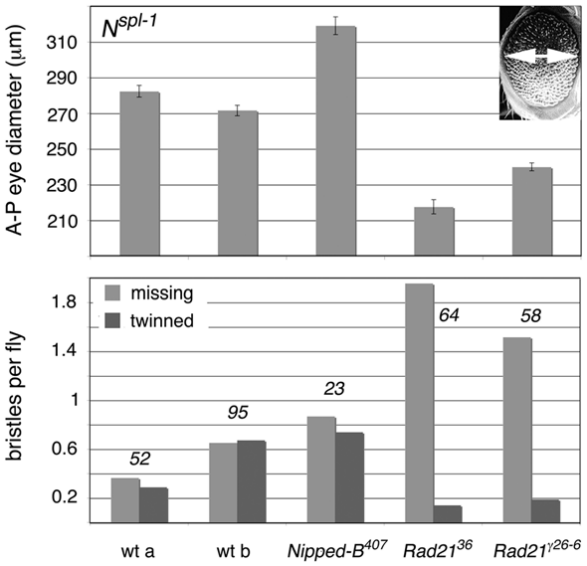
Dominant effects of *Nipped-B* and *Rad21* mutations on *Notch*-*split* (*N^spl-1^*) mutant phenotypes. The top panel compares the eye phenotype in two wild-type backgrounds (wt a, Oregon R; wt b, Canton S), to flies heterozygous for *Nipped-B^407^*, *Rad21^36^* (*vtd^36^*), and *Rad21^γ26-6^* (*vtd^γ26-6^*). Eye diameter was measured as shown in the upper right. At least 30 eyes were scored for each genotype. Error bars are standard errors. The bottom panel shows the effects of the heterozygous *Nipped-B* and *Rad21* mutations on the four scutellar macrochaete (large bristles). The number of flies scored for bristles is given above the bars.

We tested if two loss-of-function *Rad21* mutations [12], the *vtd^36^* missense mutation, and the *vtd^γ26-6^* splice site mutation, dominantly alter the *N^spl-1^* mutant phenotypes. Both increased the severity of the eye phenotype, and consistent with a previous report [9], the *Nipped-B^407^* null allele suppressed the eye phenotype (Figure 6). Both *Rad21* alleles also decreased the number of scutellar macrochaete (Figure 6). The simplest explanation is that reduced Rad21 dosage increases E(spl)-C expression in the developing eye and bristles, reducing the number of cells that adopt neural fate and become photoreceptors or bristles.

Knockdown of either Nipped-B or Rad21 increases E(spl)-C transcription in BG3 cells. Thus the opposing effects of *Nipped-B* and *Rad21* mutations on the *N^spl-1^* eye phenotype appear contradictory. We posit, however, that they reflect biphasic effects on E(spl)-C expression similar to those seen in BG3 cells (Figure 3). Heterozygous *Nipped-B* null mutations reduce *Nipped-B* mRNA by only 25% in vivo [8] and thus their suppression of *N^spl-1^* could reflect a decrease in E(spl)-C transcription caused by a biphasic effect. Although the biphasic effect is transitory with an 80% Nipped-B reduction in BG3 cells, it may last longer with a 25% reduction in vivo, and the critical phase for E(spl)-C expression in the developing eye at the morphogenetic furrow likely lasts for a much shorter time than three days [42].

### Cohesin's Effects on E(spl)-C and *invected-engrailed* Transcription in BG3 Cells are Exceptional

We measured effects of Nipped-B and Rad21 on gene expression in BG3 cells using microarrays to (a) see if the effects of cohesin on E(spl)-C and *invected-engrailed* expression are unique, (b) look for effects of cohesin on regulators of E(spl)-C and *engrailed*, and (c) obtain a comprehensive view of the role of cohesin in gene expression. We used two samples for three days after RNAi treatment, one four day and one six day sample for both Nipped-B and Rad21, and mock RNAi controls for each time point. Comparing log_2_ expression values, the genome-wide correlation coefficients between the four control samples were greater than 0.99.

**Figure 7:**
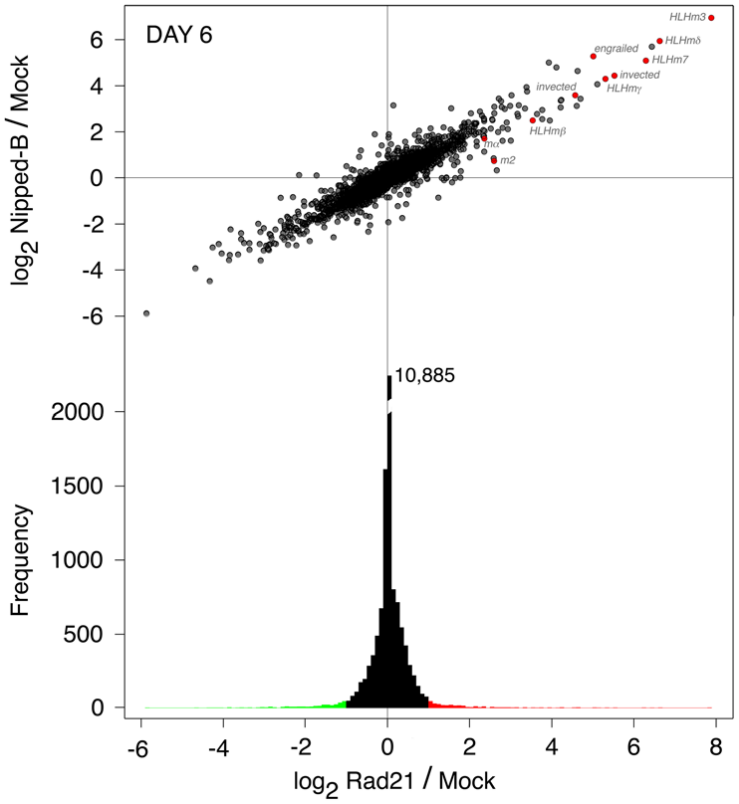
Genome-wide effects of Rad21 and Nipped-B RNAi on RNA transcripts in BG3 cells. The top graph shows the effects of Rad21 knockdown on transcript levels (log_2_ Rad21/Mock) versus the effects of Nipped-B knockdown (log_2_ Nipped-B/Mock), 6 days after RNAi for all 18,770 probes on the microarray. E(spl)-C and *invected-engrailed* transcripts are red. The bottom is an aligned histogram of the effects of Rad21 RNAi, with transcripts that increase 2-fold or more in expression in red, and transcripts that decrease 2-fold or more in green.

Strikingly, seven of the twelve transcripts that increase the most six days after Rad21 RNAi treatment are from the E(spl)-C and *invected-engrailed* (Figure 7, Figure S1, Table S4). Biphasic effects are seen, as some E(spl)-C transcripts decrease after 3 days of Nipped-B RNAi, but increase by day 6 (Figure S1, Table S4). E(spl)-C and *invected-engrailed* transcripts are present at relatively low levels in mock RNAi controls (Figure S2, Table S4). Thus the E(spl)-C and *invected-engrailed* are expressed at modest levels, and are unusually sensitive to cohesin.

Other genes located in regions of cohesin-H3K27Me3 overlap also significantly increase in expression with cohesin or Nipped-B knockdown, including *jing*, *Psc*, *Su(z)2*, *hth*, and *Lim1* (Tables S1 and S4). The increases are from 1.4 to 4-fold, and less than those observed with the E(spl)-C and *invected-engrailed*, but these genes are already expressed at 10 to 500-fold higher levels than the E(spl)-C prior to cohesin or Nipped-B knockdown, despite the extensive H3K27Me3 in their transcribed regions (Table S4). After knockdown, their expression ranges from 2-fold less to 4-fold more than E(spl)-C transcripts, suggesting that the lower fold-increases in expression of these genes with cohesin knockdown reflects their initial higher expression levels. We conclude that all genes in regions of substantial cohesin-H3K27Me3 overlap in BG3 cells are not silenced, and are negatively regulated by cohesin.

### Cohesin Knockdown Increases Expression of Notch Pathway Genes

BG3 cells are derived from central nervous system, but the proneural genes (*ac*, *sc*, *l'sc*, *ato*, *da*) that promote E(spl)-C expression [42], [43] are not expressed (Table S4). E(spl)-C genes are activated by Notch, and the genes encoding Notch (N), the Suppressor of Hairless [Su(H)] protein that tethers the Notch intracellular fragment to target genes, the Mastermind (Mam) coactivator, and both the Delta (Dl) and Serrate (Ser) Notch ligands are expressed. Cohesin RNAi increases *Ser* ligand transcripts 6-fold on day 3 and 25-fold by day 6, and thus elevated Notch signaling may help increase E(spl)-C transcription (Figure S1, Table S4).

Lack of proneural gene transcripts suggests that Notch, which alone is insufficient to activate E(spl)-C genes [42], cooperates with other unknown activators to induce E(spl)-C expression. Binding sites for many transcription factors are conserved in the E(spl)-C between *Drosophila* species [44] and some of these (*Adf1*, *broad*, *Trl*, *Eip74EF*, *dorsal*, *tramtrack*, *zeste*) are expressed in BG3 cells (Table S4).

Effects of cohesin on the Notch pathway cannot explain the effects of *Nipped-B* and *Rad21* mutations on *N^spl-1^* phenotypes described above. If *Rad21* mutations increase Notch signaling, they should increase proneural gene expression and suppress *N^spl-1^*. *Nipped-B* mutations do suppress the eye phenotype, but they have little effect on the *N^spl-1^* bristle phenotype, the *N^nd-1^* wing margin phenotype, or the *N^Ax-E2^* wing vein phenotype, indicating that they do not increase Notch signaling in vivo [9]. Thus a biphasic effect on E(spl)-C transcription remains the simplest explanation for the opposite effects of *Nipped-B* and *Rad21* mutations on the *N^spl-1^* eye phenotype.

Embryonic regulators of *engrailed* (*ftz*, *eve*, *prd*, *slp*, *odd*) are not expressed before or after cohesin RNAi (Table S4). The genes that regulate *engrailed* in later stages, however, are unknown, and thus indirect effects of cohesin RNAi on *invected-engrailed* expression cannot be ruled out. We note, however, that the modest changes in expression seen for most genes are unlikely to cause the unusually large changes in *invected* and *engrailed* expression.

### Cohesin Has Minimal Effects on PcG and trxG Genes

We considered the possibility that cohesin could regulate the E(spl)-C and *invected-engrailed* through effects on PcG or trxG gene transcription. Most of these genes, however, are not affected by cohesin RNAi (Table S4). Exceptions are an increase of 80% in *Pc* transcripts and a 2-fold increase in *Psc* expression by day 6, but this should increase silencing and reduce transcription. A few trxG transcripts (*brahma*, *osa*, *ash1*, *Trl*, *Bre1*) increase less than 2-fold. Cohesin had no significant effect on any of the 394 genes with H3K27Me3 that do not bind cohesin, most of which are not detectably expressed above background levels, including all the genes in the bithorax and Antennapedia complexes (Table S4).

### Cohesin Directly Regulates Gene Expression

The genome-wide effects of Nipped-B and Rad21 RNAi on gene expression after six days were very similar, with a correlation between the log_2_ Nipped-B/control and log_2_ Rad21/control expression ratios of 0.93 (Figure 7). Thus, with very few exceptions, Nipped-B and cohesin regulate the same genes to similar extents. Genome-wide, slightly more than 10% of transcripts showed statistically significant changes in one or more RNAi treatments, with 959 transcripts increasing, and 1025 decreasing (Figure S3).

Comparison of the effects of cohesin on transcripts to its binding pattern in BG3 cells argues that many of the effects of Nipped-B and cohesin on gene expression are direct. To ensure that we examined genes that respond consistently, we analyzed transcripts that showed 2-fold or greater increases or decreases in two or more RNAi treatments. By these criteria, 340 transcripts increase, and 414 decrease. 333 of the up-regulated and 407 of the down-regulated genes are euchromatic, allowing us to determine cohesin and RNA polymerase (PolII) binding from chromatin immunoprecipitation data.

Justified by their genome-wide co-localization [15], we combined the ChIP-chip data for Nipped-B and Smc1 and identified the genes in which these proteins bind within the transcription units at p≤10^−3^. By these criteria, 57% (189/333) of the genes that increase, and 36% (146/407) of the genes that decrease in expression bind cohesin (Table S5), which is a significant difference (p = 9.7×10^−9^). PolII binding does not differ, with binding to 68% (225/333) of the increasing and 66% (268/407) of the decreasing genes (Table S5). It is not unexpected that PolII binding is not detected in some cases because many genes are expressed at low levels and have low polymerase density. PolII binding is detected more frequently with the cohesin-binding genes, in 83% of the increasing and 82% of the decreasing genes (Table S5). We conclude that more genes that increase in expression with cohesin RNAi bind cohesin compared to genes that decrease.

Both increasing and decreasing genes bind cohesin at a higher than average frequency. Genome-wide, 19% (816/4282) of PolII-binding genes also bind cohesin, compared to 70% (157/225) of the PolII-binding genes that increase in expression, and 45% (120/268) of the PolII-binding genes that decrease (Table S5). This argues that cohesin directly affects expression, and that negative effects are more common than positive. These data also indicate that many changes in expression that occur with cohesin RNAi are indirect.

Analysis of cohesin-binding genes further argues that the large increases in E(spl)-C and *invected-engrailed* transcripts that occur with cohesin knockdown are unique. Of the 816 genes in BG3 cells that bind both cohesin and PolII, 804 are detected by the expression microarray. 341 (42%) of these increase in expression by 20% or more with Rad21 knockdown, and 136 (17%) decrease 20% or more (Figure S4). 54 (7%) are not detectably expressed, and 273 (34%) change less than 20% in expression (Figure S4). For genes that increase 20% or more, the median increase is 50%. For the genes that decrease 20% or more, the median decrease is 35%. Thus the effect on expression of most cohesin-binding genes is less than 2-fold.

### Cohesin Has Minor Effects on Genes Involved in Translation and Cell Division

The top gene ontology (GO) categories for genes that increase in expression with cohesin RNAi involve development, while the top categories for decreasing transcripts involve protein translation (Figure S3, Table S6). All ribosomal protein transcripts decrease an average of 15%, and all aminoacyl tRNA synthetase transcripts decrease an average of 33% (Table S4). The most significant cell division category is mitotic spindle elongation (Table S6), but most genes in this case encode ribosomal proteins. There are slight increases, all less than 2-fold, in transcripts for *cyclin B*, some cohesion factors and condensin subunits, consistent with a mild G2/M delay [33].

## Discussion

### Cohesin Regulates the *Enhancer of split* and *invected-engrailed* Gene Complexes in a Cell-Specific Manner

Here we show that in BG3 cells derived from central nervous system, the E(spl)-C, and the complex containing *invected* and *engrailed* share exceptional attributes: (a) cohesin binds over the entire gene complex and not just to individual genes, (b) cohesin binds throughout a large H3K27Me3 domain, and (c) they show unusually large increases in transcription when cohesin is reduced. We posit, therefore, that cohesin directly regulates these gene complexes.

This is supported by the contrasts in histone modification, cohesin binding, and the response to cohesin between BG3 and Sg4 cells. In Sg4 cells, cohesin binds only three of the active E(spl)-C genes, there is no H3K27Me3, and expression not substantially affected by cohesin. Thus the effect of cohesin on the E(spl)-C correlates with presence of cohesin and H3K27Me3 domains. The *invected-engrailed* complex in Sg4 cells shows the typical pattern for PcG silenced genes. It is coated by H3K27Me3, there is no cohesin, and it is silent before or after cohesin RNAi. Thus, we suggest that in BG3 cells, cohesin prevents complete silencing of *invected* and *engrailed* by PcG proteins, and/or that lack of silencing promotes cohesin binding. This latter possibility alone seems unlikely, given that many non-silenced and active genes do not bind cohesin, and that cohesin domains that extend over entire gene complexes are rare. For instance, only selected active E(spl)-C genes bind cohesin in Sg4 cells, in which there is no H3K27Me3, but the entire complex binds cohesin in BG3 cells, when it is also coated by H3K27Me3, indicating that lack of silencing or gene expression by itself is insufficient to establish the cohesin domain. We currently do not know the factors that determine when and where a cohesin domain is established.

**Figure 8:**
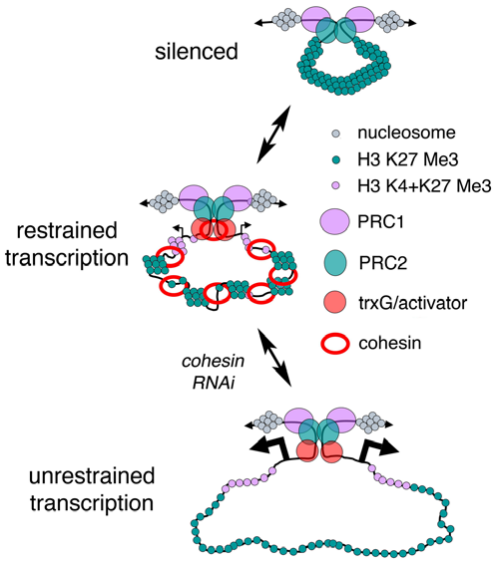
Speculative model for regulation of gene complexes by cohesin. The top depicts a PcG-silenced complex contained in a loop created by PRE-PRE interactions. There is little or no transcription, and we posit that the silenced chromatin diameter prevents encirclement by cohesin. The nucleosomes have trimethylation of histone H3 on lysine 27 (green). The middle diagram depicts a gene complex in which cohesin, trithorax group (trxG), transcriptional activators, and PcG proteins combine to create an intermediate chromatin structure with aspects of both silenced and active regions that permits modest transcription (angled arrows); nucleosomes near the transcription start sites also have trimethylation of histone H3 on lysine 4 (pink). Based on the biphasic effects of Nipped-B and cohesin knockdown on some E(spl)-C transcripts, we posit that when cohesin levels are reduced, the chromatin structure first becomes closer to the silenced state, decreasing transcription, and that the higher order structure associated with silencing is eventually lost, leading to unrestrained transcription (bottom).

The similarities in chromatin structure and hypersensitivity to cohesin between the E(spl)-C and *invected-engrailed* complexes in BG3 cells lead us to speculate that in cases of cohesin and H3K27Me3 overlap, cohesin helps create an intermediate chromatin structure with aspects of both silenced and active regions (Figure 8). Such a dual role is consistent with the biphasic effects of Nipped-B and Rad21 RNAi on E(spl)-C transcription. When cohesin levels are reduced, silencing becomes temporarily stronger, but eventually a specific chromatin structure needed to repress transcription is lost, leading to overexpression. In other regions of cohesin-H3K27Me3 overlap, where genes such as *Psc* and *hth* are expressed at higher levels, the structural balance favors the active state. RNA levels are still increased in these cases by reducing cohesin levels, however, indicating that transcription is still restricted. At present, we do not know if cohesin binding is reduced selectively at specific sites when cohesin or Nipped-B dosage is only slightly reduced, which might contribute to biphasic effects at some genes. The lack of an effect of CP190 insulator protein on E(spl)-C and *invected-engrailed* expression argues against the possibility that changes in insulator activity contribute to the changes in E(spl)-C and *invected-engrailed* transcription that occur with cohesin knockdown.

In *S. cerevisiae*, cohesin inhibits spreading of SIR silencing proteins and establishment of silencing [45], [46], suggesting that cohesin might have a similar effect on PcG function at the E(spl)-C and *invected-engrailed* complex. Cohesin binds the silent *HMR* mating type locus [47], [48], where it helps form a chromatin boundary [45], and mediate sister cohesion [49], [50]. It remains to be determined if cohesin's functions at *HMR* are analogous to its roles at E(spl)-C or *invected-engrailed*, but we note that the H3K27Me3 mark at *invected-engrailed* extends far beyond the cohesin domain at one end, arguing that cohesin does not form a chromatin boundary.

The finding that H3K27Me3 coats the E(spl)-C and *invected-engrailed* complex in BG3 cells, and that many of the genes in these two complexes bind PolII, raises the question if they are equivalent to bivalent genes in mammals. Including the E(spl)-C and *invected-engrailed*, and the five other genes in regions of cohesin-H3K27Me3 overlap, only 13% of the 480 genes marked by H3K27Me3 in BG3 cells bind PolII, and the vast majority of marked genes are not detectably expressed above background levels. Bivalent genes are defined by the simultaneous presence of the H3K27Me3 mark made by E(z) orthologs at silenced genes, and the histone H3 lysine 4 trimethylation (H3K4Me3) modification made by Trithorax orthologs at active genes [51]–[53]. Bivalent genes are frequent in embryonic stem cells, but also occur in lineage-restricted cells [53]. Like the E(spl)-C and *invected-engrailed* complex in BG3 cells, many bivalent genes encode transcription factors and are expressed at modest levels [52], [54]. The *invected-engrailed* complex in BG3 cells has both H3K4Me3 and H3K27Me3 modifications, but the E(spl)-C shows only a little H3K4Me3 (Y.B. Schwartz, T.G. Kahn, P. Stenberg, K. Ohno, R. Bourgon, V. Pirrotta, submitted). Thus *invected-engrailed* matches the original definition of bivalent genes.

### Does Cohesin Regulate the E(spl)-C and *invected-engrailed* In Vivo?

The enhancement of *N^spl-1^* mutant phenotypes by *Rad21* (*vtd*) mutations reported here supports the idea that cohesin restricts E(spl)-C transcription during eye and bristle development, because these are the phenotypic changes seen when E(spl)-C activity is increased by gene duplication, forced overexpression, or hypermorphic mutations, and opposite of what is caused by an increase in Notch signaling or decrease in E(spl)-C dosage [37]–[41].

Heterozygous *Nipped-B* mutations suppress the *N^spl-1^* eye phenotype, suggesting that they either reduce E(spl)-C expression or increase Notch signaling. Because heterozygous *Nipped-B* null mutations only reduce *Nipped-B* mRNA by 25% [8], this is consistent with an in vivo biphasic effect on E(spl)-C transcription similar to that seen in BG3 cells. Based on the genome-wide analysis in BG3 cells, which shows that Nipped-B and cohesin regulate the same genes to similar extents, it is unlikely that Nipped-B and Rad21 have opposing effects on eye development by regulating different genes. Also, *Nipped-B* mutations do not affect other sensitive *Notch* mutant phenotypes, arguing that the effect on *N^spl-1^* is not through increasing Notch signaling [9]. Given the essential nature of cohesin in cell division, and the complex spatial and temporal pattern of E(spl)-C expression in vivo, it will not be simple to confirm that Nipped-B and cohesin directly affect the levels of specific E(spl)-C transcripts in vivo, or rule out potential indirect effects. Indeed, given the contrast in binding of cohesin to the E(spl)-C between BG3 and Sg4 cells, in vivo effects of cohesin likely occur in only a select population of E(spl)-C expressing cells.

For similar reasons, it will also not be straightforward to confirm that PcG proteins regulate the E(spl)-C in vivo. Effects of PcG on E(spl)-C function have not been reported, and genome-wide mapping in other cell lines, whole organisms, or imaginal discs has not revealed that the E(spl)-C gene is a PcG target [30], [31], [55]–[57]. Nonetheless, the H3K27Me3 pattern and the effects of Pc knockdown on E(spl)-C expression in BG3 cells argue strongly that E(spl)-C is a PcG target, although this may occur only in a small fraction of cells in vivo.

It is unknown if *invected* and *engrailed* are regulated by cohesin in vivo. Our results suggest that this may occur in cells in which *engrailed* is active, but partially repressed by PcG proteins, such as the posterior compartment of the wing imaginal disc [58]. No dominant effects of *Nipped-B* or cohesin mutations on compartment formation have been observed in otherwise wild-type flies, but the feedback loop at the wing anterior-posterior boundary that controls *engrailed, hedgehog, patched, wingless* and *decapentaplegic* expression [59] may prevent or counteract increases in *engrailed* expression. The feedback mechanisms may be unbalanced in *hedgehog^Moonrat^* mutants, in which ectopic *hedgehog* expression in the anterior compartment causes overgrowth [60], [61]. *Rad21* (*vtd*) and *Nipped-B* mutations dominantly suppress this overgrowth [12], [62], and one possibility is that increased *engrailed* expression helps restore the autoregulatory loop.

### Do Genes Hypersensitive to Cohesin Contribute to Cornelia de Lange Syndrome (CdLS)?

Heterozygous loss-of-function mutations in the *Nipped-B-Like* (*NIPBL*) ortholog of *Nipped-B* cause CdLS, characterized by slow growth, mental retardation, autistic features, craniofacial abnormalities, and structural defects in limbs, gut, heart and kidney [63], [64]. Mutations that change amino acid residues in the Smc1 or Smc3 cohesin subunits cause milder CdLS [65], [66]. Cells from CdLS individuals do not have significant defects in chromatid cohesion [67]–[69], and *NIPBL* mRNA is only reduced by 15 to 30% in cells from CdLS individuals [70], [71], indicating that the developmental deficits arise from changes in gene expression.

Relative to healthy controls, over a thousand genes are differentially expressed in CdLS lymphocyte cell lines with *NIPBL* mutations or mutant Smc1 [71]. As with cohesin knockdown in Drosophila BG3 cells, some genes increase in expression and some decrease. Most changes in lymphocytes, however, are less than 2-fold, and the largest effect is less than 4-fold. It is unknown if lymphocytes contain significant overlaps of cohesin and H3K27Me3, and therefore whether or not they might have hypersensitive genes similar to those in BG3 cells. Given the small reductions in cohesion factor activity that cause CdLS, the findings in BG3 cells suggest that genes that are hypersensitive to cohesin in only a subset of cells are the most likely to be strongly affected, and significantly alter development.

## Materials and Methods

### Cell Culture and RNAi

BG3 cells were cultured in Schneider's media with 10% FCS and 10 µg per ml insulin. Sg4 cells were grown in Schneider's containing 10% FCS. For RNAi, cells were plated at 5×10^6^ cells per 3 cm well for BG3 cells, and 3×10^6^ for Sg4 cells. Media was replaced with 1 ml of Express Five SFM (Invitrogen) with 1% FCS, (and 10 µg per ml insulin for BG3 cells). For cohesion factors and Polycomb, from 0.7 to 40 µg of dsRNA was added per well, and 80 µg was used for CP190 knockdown. Media was adjusted to 3 ml and 10% FCS with Schneider's media after 2 hrs. Cells were replated as needed. Templates for dsRNA synthesis were made by PCR from cDNA or genomic DNA templates using primers with T7 promoters (Table S7). In most experiments, equal amounts of two dsRNAs against each target were used. Both individual dsRNAs knocked down the targets, but knockdown was generally more efficient with a mixture. All dsRNA sequences were scanned against the genome to avoid off-target effects. To determine transcript half-lives, actinomycin D was added to cultures at 5 µg per ml, RNA was extracted every 30 min up to 2 hours, and half-lives were calculated assuming exponential decay.

### RNA Quantification

Total RNA was isolated using Trizol (Invitrogen), treated with DNase I (Epicentre), chloroform extracted, ethanol precipitated and dissolved in water. cDNA was synthesized using random hexamer primers and SuperScript VILO reverse transcriptase (Invitrogen). Transcripts were quantified using Sybr green real-time PCR (Clontech) and gene-specific primers (Table S8) calibrated with genomic DNA. RNA levels were calculated adjusting for amplification efficiency [72] and normalizing to internal *RpL32* transcripts and external genomic DNA standards. Standard errors of the mean were calculated using all PCR replicates from all biological replicates.

### Protein Extracts and Western Blots

Cells were washed in phosphate-buffered saline (PBS), lysed in RIPA buffer (5 µl per 10^6^ cells), insoluble material removed by centrifugation, and extracts were stored at −80°. Nipped-B, Smc1, SA, Rad21, Polycomb, and CP190 proteins were quantified by SDS-PAGE western blots using chemiluminescence imaging with Actin as a standard and previously described antisera [6], [7], [15], [20], [73].

### Metaphase Spreads

Cells (3×10^6^) were incubated in media with 3 mg per ml colchicines for 4 hr, washed in PBS, suspended in hypotonic (1% sodium citrate) for 4 min, collected by centrifugation, suspended in 0.1 ml hypotonic and fixed with 1 ml ice-cold methanol:acetic acid (3∶1). Fixed cells were suspended in 60 µl of methanol:acetic acid, dropped onto a microscope slide from a distance of 50 to 60 cm, and covered with a coverglass. Slides were frozen on dry ice for 20 min, and rinsed with PBST (PBS with 1% Triton X-100) 3 times after removing the coverslip. Chromosomes were stained with 0.5 µg DAPI per ml in PBS for 10 min, rinsed with PBST, mounted in BioRad FluoroGard, and observed by fluorescence microscopy.

### Effects of *Nipped-B* and *Rad21* (*vtd*) Mutations on *N^spl-1^* Mutant Phenotypes

*w^a^ N^spl-1^* females were crossed to wild-type males or males with *Nipped-B* and *vtd* mutations over balancers with dominant markers at 25°. The anterior-posterior diameter of the eyes of male progeny were measured with a reticule in a dissection microscope, and scutellar macrochaete were counted.

### Genome-Wide Transcript Analysis

Five µg of total RNA purified by Qiagen RNeasy minicolumns was used to make cRNA probes using Affymetrix GeneChip HT One-Cycle Target Labeling and Controls Kit according to the manufacturer's instructions. Probes were hybridized to Affymetrix GeneChip *Drosophila* Genome 2.0 arrays, processed and scanned using Affymetrix procedures. Quality metrics for each array were monitored by spike-in labeling controls and hybridization/staining controls using Microarray Suite 5.0 (MAS5) algorithms from GeneChip® Operating Software v1.4, (GCOS) (Affymetrix, Inc). Probe cell intensities for each array were normalized using GCRMA algorithms, which consist of background adjustment and quantile normalization, accounting for probe GC content [74]. Normalization was executed using the R statistical environment [R Foundation for Statistical Computing, Vienna, 2007; ISBN 3-900051-07-0; www.R-project.org] and the Bioconductor package (www.bioconductor.org) [75]. Transcript levels from Rad21 and Nipped-B RNAi treatments were compared to those of mock RNAi controls at 3 and >3 days (4 and 6 days) (N = 4 per RNAi comparison; N = 2 per treatment condition). A balanced 2-way ANOVA was performed on GCRMA-normalized log_2_ signal intensities to assess expression variability with regard to RNAi treatment (FDR≤0.1) [76], [77]. Differentially expressed groups were analyzed for gene ontology enrichment using Fisher's exact test in the GOEAST package [78]. The data are available in the GEO database (accession no. GSE16152).

### Correlation of Chromatin Immunoprecipitation and Gene Expression Data

The Nipped-B, Smc1, RNA polymerase II, H3K27Me3 and control cel files for BG3 and Sg4 cell chromatin immunoprecipitations (GEO acc. no. GSE9248; ArrayExpress acc. no. E-MEXP-535) were processed using MAT [79] to generate cohesin-Nipped-B, H3K27Me3, and PolII bed files at p≤10^−3^ that were visualized using the Affymetrix Integrated Genome Browser. Transcription units that overlap cohesin-Nipped-B, H3K27Me3, and PolII binding regions were identified using the April 2006 genome annotations [80].

## Supporting Information

Table S1 (0.05 MB DOC)

Table S2 (0.03 MB DOC)

Table S3 (0.06 MB DOC)

Table S4 (6.48 MB XLS)

Table S5 (0.05 MB DOC)

Table S6 (0.91 MB XLS)

Table S7 (0.04 MB DOC)

Table S8 (0.05 MB DOC)

Figure S1 Time courses for the twelve transcripts most increased by Rad21 knockdown in BG3 cells. Transcripts are shown in descending order from the left. The fold-changes in transcript levels with Rad21 (black) and Nipped-B (red) knockdown on days 3, 4 and 6 are shown for each transcript as the log2 RNAi/Mock ratio. Seven mRNAs from the E(spl)-C and the *invected-engrailed* complex are indicated with asterisks. The *invected* gene is represented by two probes. Three E(spl)-C genes (*HLHm3*, *HLHm7*, *HLHmγ*) show biphasic changes with Nipped-B knockdown, decreasing on day 3, but increasing by day 6.
(0.63 MB TIF)

Figure S2 Relative expression of genes altered in expression by Rad21 RNAi in BG3 cells. The top graph plots the control Mock expression level versus the fold-change in expression with Rad21 knockdown for all 18,770 probes, and the aligned histogram distribution at the bottom shows the number of transcripts at each expression level in the Mock RNAi control cells. The genes that increase 2-fold or more in expression are in red, and the genes that decrease 2-fold or more are green. The strongly affected E(spl)-C and *invected-engrailed* complex transcripts are labeled.
(0.63 MB TIF)

Figure S3 Effects of Rad21 and Nipped-B RNAi on gene expression in BG3 cells. The heat maps show the changes in expression for the most significant biological function gene ontology (GO) categories for the genes that increase in expression (developmental process) and that decrease in expression with cohesin knockdown (translation). The bottom panel shows the heat map for all 959 genes that show significant increases in expression and all 1025 genes that show significant decreases in expression with Rad21 and Nipped-B RNAi knockdown. The significant GO categories for the affected genes are listed in Table S6, with the probe identities in each group.
(1.01 MB TIF)

Figure S4 Effects of Rad21 on genes binding both cohesin and RNA polymerase II in BG3 cells. The 804 genes binding both PolII and cohesin whose expression was measured by the microarray are broken into four categories based on their response to Rad21 knockdown after six days, with the number of genes in each category indicated on the pie chart.
(0.34 MB TIF)
