# Supplementary material for: Regulation of the Drosophila Enhancer of split and invected-engrailed Gene Complexes by Sister Chromatid Cohesion Proteins
Source: PLoS One. 2009 Jul 9;4(7):e6202. doi: 10.1371/journal.pone.0006202 (PMC2703808; doi:10.1371/journal.pone.0006202)
Supplement: Table S1 — (0.05 MB DOC) [file pone.0006202.s001.doc]

**Table S1. Regions of cohesin – H3K27Me3 overlap.**

| Region^a^ | Size | Genes | Cell Type | Effect of cohesin RNAi^b^ |
| --- | --- | --- | --- | --- |
| 2L 14,531,380 – 14,586,447 | 55.1 kb | - | BG3 | - |
| 2R 2,390,180 – 2,434,731 | 44.6 | *jing* | BG3 | 1.4-fold increase |
| 2R 7,361,191 – 7,416,761 | 55.6 | *invected*, *engrailed* | BG3 | 24-fold increase |
| 2R 8,849,716 – 8,930,632 | 80.9 | *Psc, Su(z)2* | BG3, Sg4 | 2-fold increase |
| 3R 6,445,227 – 6,450,047 | 4.8 | *hth* | BG3 | 4-fold increase |
| 3R 11,472,282 – 11,481,587 | 9.3 | - | BG3 | - |
| 3R 21,822,901 – 21,866,784 | 43.9 | E(spl)-C | BG3 | 235-fold increase |
| 3R 26,597,168 – 26,602,515 | 5.3 | *zfh1* | Sg4 | - |
| X 8,662,774 – 8,696,222 | 33.4 | *Lim1* | BG3 | 2-fold increase |

^a^Regions of overlap > 2 kb at p ≤ 10^-3^ with product of MAT scores > 50; positions are indicated by chromosome arm and nucleotide numbers from the April 2006 release of the *Drosophila* genome sequence.

^b^Maximal effect on transcript levels for any gene in region seen in BG3 expression microarray analysis with Rad21 or Nipped-B RNAi knockdown.
