# Supplementary material for: Regulation of the Drosophila Enhancer of split and invected-engrailed Gene Complexes by Sister Chromatid Cohesion Proteins
Source: PLoS One. 2009 Jul 9;4(7):e6202. doi: 10.1371/journal.pone.0006202 (PMC2703808; doi:10.1371/journal.pone.0006202)
Supplement: Table S2 — (0.03 MB DOC) [file pone.0006202.s002.doc]

**Table S2. Half-lives of E(spl)-C transcripts.**

| Gene | Rad21/Mock^a^ | Mock t_1/2_ (min)^b^ | Rad21 t_1/2_ (min)^c^ |
| --- | --- | --- | --- |
| *HLHmδ* | 10.2 | 24.5 | 21.4 |
| *HLHmγ* | 1.9 | 16.7 | 18.2 |
| *mα* | 1.3 | 15.6 | 15.5 |
| *HLHm3* | 1.7 | 23.4 | 24.8 |
| *HLHm7* | 6.3 | 60 | 65 |

^a^Fold-increase in transcript level in Rad21 RNAi-treated cells 3 days after treatment.

^b^Half-life of transcript in mock-treated cells after Actinomycin D.

^c^Half-life of transcript in Rad21 RNAi-treated cells after Actinomycin D.
