# Supplementary material for: Regulation of the Drosophila Enhancer of split and invected-engrailed Gene Complexes by Sister Chromatid Cohesion Proteins
Source: PLoS One. 2009 Jul 9;4(7):e6202. doi: 10.1371/journal.pone.0006202 (PMC2703808; doi:10.1371/journal.pone.0006202)
Supplement: Table S3 — (0.06 MB DOC) [file pone.0006202.s003.doc]

**Table S3. Effects of Rad21 and Nipped-B RNAi on precocious sister chromatid separation (PSCS) and hyperploidy.**

| Cell | RNAi | # Cells | # Hyperploid^a^ | % Hyperploid | p value Hyperploid^b^ | # Chromosomes^c^ | # PSCS Chromosomes | % PSCS Chromosomes | p value PSCS^d^ |
| --- | --- | --- | --- | --- | --- | --- | --- | --- | --- |
| BG3 | Mock | 71 | 3 | 4.2 | -- | 322 | 28 | 9 | -- |
| BG3 | Rad21 | 30 | 1 | 3.3 | 0.76 | 146 | 35 | 24 | 1.4x10^-5^ |
| BG3 | Nipped-B | 31 | 1 | 3.2 | 0.77 | 133 | 43 | 32 | 1.7x10^-9^ |
| Sg4 | Mock | 74 | 3 | 4.1 | -- | 610 | 142 | 23 | -- |
| Sg4 | Rad21 | 37 | 2 | 5.4 | 0.54 | 249 | 118 | 47 | 6.6x10^-12^ |
| Sg4 | Nipped-B | 37 | 4 | 11 | 0.17 | 310 | 94 | 30 | 1.3x10^-2^ |

^a^BG3 cells are diploid male with four large autosomes, two 4th dot chromosomes, one X and one Y chromosome. Sg4 cells are partially tetraploid, with eight large autosomes, two 4th dot chromosomes, and two X chromosomes. Cells with one or more extra large chromosomes were scored as hyperploid.

^b^Comparison of RNAi treated to Mock by Fisher’s exact test.

^c^Only X chromosomes and large autosomes with clear morphology were scored for PSCS.

^d^Comparison of RNAi treated to Mock by Fisher’s exact test.
