# Supplementary material for: Regulation of the Drosophila Enhancer of split and invected-engrailed Gene Complexes by Sister Chromatid Cohesion Proteins
Source: PLoS One. 2009 Jul 9;4(7):e6202. doi: 10.1371/journal.pone.0006202 (PMC2703808; doi:10.1371/journal.pone.0006202)
Supplement: Table S5 — (0.05 MB DOC) [file pone.0006202.s005.doc]

**Table S5. RNA polymerase II and cohesin binding to genes that increase or decrease in expression with Rad21 or Nipped-B RNAi.**

| Expression Change^a^ | Genes (G)^b^ | PoIII (P)^c^ | P/G | cohesin (C)^d^ | | C/G | | P+C^e^ | (P+C)/C | (P+C)/P |
| --- | --- | --- | --- | --- | --- | --- | --- | --- | --- | --- |
| All |  | 4282 |  |  | |  | | 816 |  | 0.19 |
| Increase | 333 | 225 | 0.68 | 189 | | 0.57 | | 157 | 0.83 | 0.70 |
| Decrease | 407 | 268 | 0.66 | 146 | | 0.36 | | 120 | 0.82 | 0.45 |
| Increase vs Decrease^f^ |  |  | p = 0.34 |  | p = 9.7x10^-9^ | | |  |  |  |
| Increase vs All^g^ |  |  |  |  | | |  |  |  | p = 2.4 x 10^-57^ |
| Decrease vs All^g^ |  |  |  |  | | |  |  |  | p = 2.3 x 10^-20^ |

^a^All genes, or genes that increase or decrease in expression ≥ 2-fold in two or more RNAi treatments

^b^Number of genes (G) with indicated expression change

^c^Number of genes with indicated expression change that bind RNA polymerase II (PolII, P)

^d^Number of genes with indicated expression change that bind cohesin and Nipped-B (C)

^e^Number of genes with indicated expression change that bind both PolII (P) and cohesin (C)

^f^Comparison for PolII or cohesin binding with Fisher’s exact test

^g^Comparison of PolII-binding genes for cohesin binding with Fisher’s exact test
