# Supplementary material for: Regulation of the Drosophila Enhancer of split and invected-engrailed Gene Complexes by Sister Chromatid Cohesion Proteins
Source: PLoS One. 2009 Jul 9;4(7):e6202. doi: 10.1371/journal.pone.0006202 (PMC2703808; doi:10.1371/journal.pone.0006202)
Supplement: Table S7 — (0.04 MB DOC) [file pone.0006202.s007.doc]

**Table S7. PCR primers for making RNAi templates.**

| Target | Forward | Reverse |
| --- | --- | --- |
| Nipped-B | TAATACGACTCACTATAGGGAGATTCGCTGTTGGGAACTATGCTGG | TAATACGACTCACTATAGGGAGATGTCGGTATCACTTTCATCGCACG |
| Nipped-B | TAATACGACTCACTATAGGGAGAGTTCAATAGCCAACGACGCCG | TAATACGACTCACTATAGGGAGATGGTCCACGACTCGCATAACCTC |
| Rad21 | TAATACGACTCACTATAGGGAGACTGGTTGGCAGCACATTGGG | TAATACGACTCACTATAGGGAGAGCATATCAGCATGGGCGTCC |
| Rad21 | TAATACGACTCACTATAGGGAGATGGGTGACGATTTTAATCAAGGAG | TAATACGACTCACTATAGGGAGACGCCTGTTTTCTGGAATTTCCTG |
| SA | TAATACGACTCACTATAGGGAGAGGGACACCACGAGCGGATA | TAATACGACTCACTATAGGGAGAGCCGTCATCAACTTCATGGC |
| SA | TAATACGACTCACTATAGGGAGATGACGCTCCTTTTGAGCCTG | TAATACGACTCACTATAGGGAGATCTCGACGTTTACGTGTGTAGGC |
| Pc | TAATACGACTCACTATAGGGCAAAGCCGAGGTGCTCAAG | TAATACGACTCACTATAGGGACGAATCGCCTTTCATGTCG |
| CP190 | TAATACGACTCACTATAGGGAGATAAACGGACGACCCATTAGC | TAATACGACTCACTATAGGGAGATTATGTCCGAAAGGATTCGC |
