# Supplementary material for: Regulation of the Drosophila Enhancer of split and invected-engrailed Gene Complexes by Sister Chromatid Cohesion Proteins
Source: PLoS One. 2009 Jul 9;4(7):e6202. doi: 10.1371/journal.pone.0006202 (PMC2703808; doi:10.1371/journal.pone.0006202)
Supplement: Table S8 — (0.05 MB DOC) [file pone.0006202.s008.doc]

**Table S8. Primers for RT-PCR.**

| Gene | Forward | Reverse |
| --- | --- | --- |
| *invected* | TTGGTCGGCGGTTCGTAACAGC | TGGGTTGGGTGATAAACTTGTCG |
| *engrailed* | TTCCACAATCAGACGCACACC | CGTATCATCCACATCCACATCAATG |
| *Abd-B* | TCCGCAAACAAGAAGACACACTCC | GGTATCAAAGGACACGACACGACG |
| *HLHmγ* | AATCAACAAGTGCCTGGACGAG | GCAAATGGGTGACGGTAAGTTC |
| *HLHmδ* | GCAAATGGGTGACGGTAAGTTC | TCCTTGAGTTCGTCCAGATACAGG |
| *HLHmβ* | CACAGAGTCTCCGAGTCCGAATC | CCAGAACCATTTTGTTGTAGTTTGG |
| *mα* | GGAGGACGAGGAGGATGTCTATG | GACTGGCTGAAGGTTGGTGGTC |
| *m1* | AGAACGCATTCGTCTGTAAAAACC | TGGGGCAAAAAGTTGGACAAGC |
| *m2* | CAAGTCAACGCCAGAGGAGTCTATC | CGCTGCTAATCAATGTGGGTGTG |
| *HLHm3* | AGGGAGTAGTGGCTGGTGTTGG | CTCATCGGTTTGCTGTGTCTGC |
| *m4* | ACCGTTCCCGTTCACTTCGTCC | ATAGCGATGGCGTTGGAGGTGCTG |
| *HLHm5* | TTGGACACCTTGAAGACCTTGG | CTGCTGCTTGACGACCTGTTTG |
| *m6* | CCGACAGTCAGCGATACGATAGC | CCTCCAATCCCACTTGAGTTGC |
| *HLHm7* | AGTGGATGTGGCTTTTGGAACC | GACGATACTGAGTGGAGTGTTGACG |
| *E(spl)m8* | ATGAACAAGTGCCTGGACAACC | CTTCCTGAGCCACCTTCTTTGG |
| *RpL32* | ATCGGTTACGGATCGAACAAGC | GTTCTGCATGAGCAGGACCTCC |
