# Supplementary figures and images for: Regulation of the Drosophila Enhancer of split and invected-engrailed Gene Complexes by Sister Chromatid Cohesion Proteins
Source: PLoS One. 2009 Jul 9;4(7):e6202. doi: 10.1371/journal.pone.0006202 (PMC2703808; doi:10.1371/journal.pone.0006202)

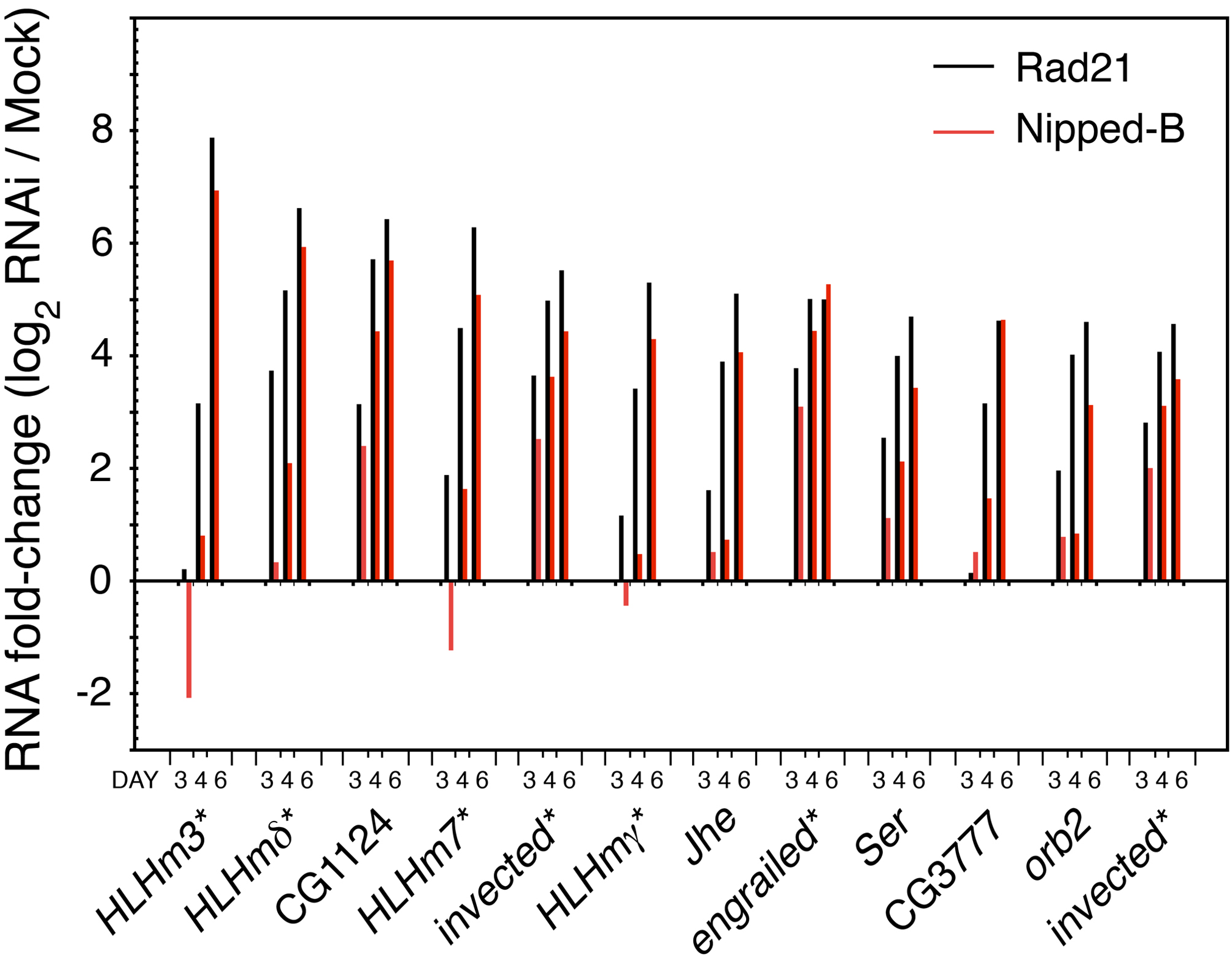

Supplement: Figure S1 — Time courses for the twelve transcripts most increased by Rad21 knockdown in BG3 cells. Transcripts are shown in descending order from the left. The fold-changes in transcript levels with Rad21 (black) and Nipped-B (red) knockdown on days 3, 4 and 6 are shown for each transcript as the log2 RNAi/Mock ratio. Seven mRNAs from the E(spl)-C and the invected-engrailed complex are indicated with asterisks. The invected gene is represented by two probes. Three E(spl)-C genes (HLHm3, HLHm7, HLHmγ) show biphasic changes with Nipped-B knockdown, decreasing on day 3, but increasing by day 6. (0.63 MB TIF) [file pone.0006202.s009.tif]

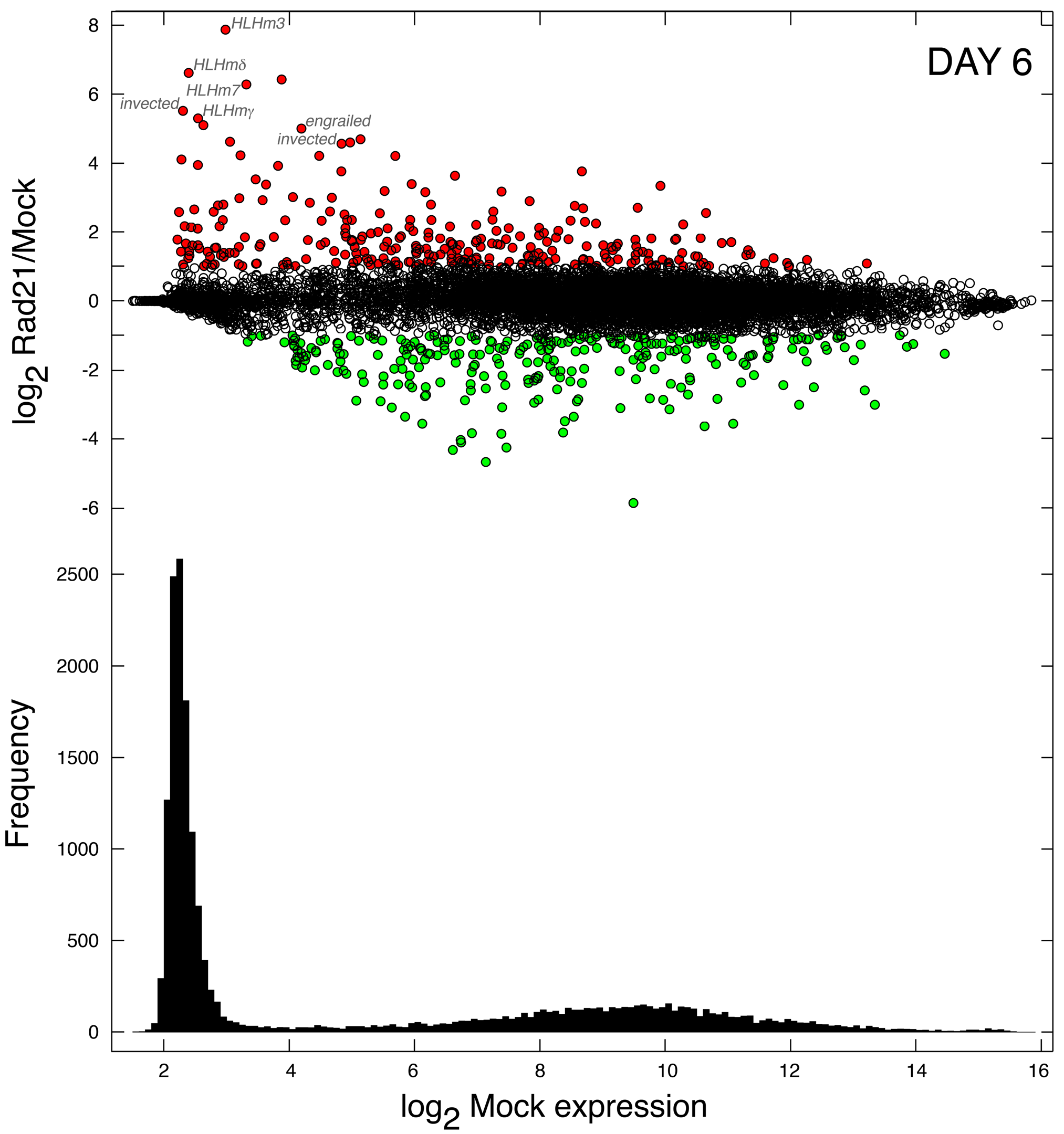

Supplement: Figure S2 — Relative expression of genes altered in expression by Rad21 RNAi in BG3 cells. The top graph plots the control Mock expression level versus the fold-change in expression with Rad21 knockdown for all 18,770 probes, and the aligned histogram distribution at the bottom shows the number of transcripts at each expression level in the Mock RNAi control cells. The genes that increase 2-fold or more in expression are in red, and the genes that decrease 2-fold or more are green. The strongly affected E(spl)-C and invected-engrailed complex transcripts are labeled. (0.63 MB TIF) [file pone.0006202.s010.tif]

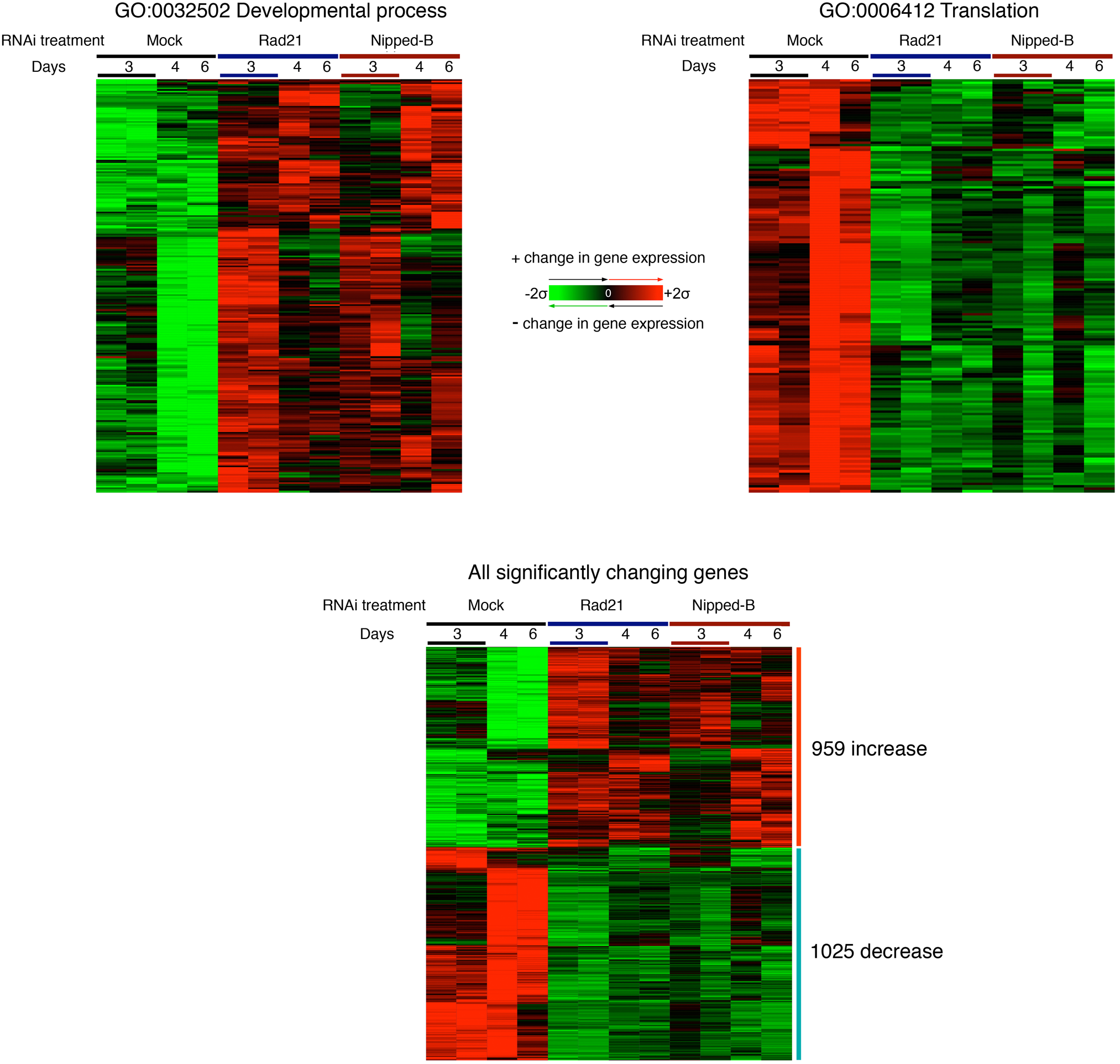

Supplement: Figure S3 — Effects of Rad21 and Nipped-B RNAi on gene expression in BG3 cells. The heat maps show the changes in expression for the most significant biological function gene ontology (GO) categories for the genes that increase in expression (developmental process) and that decrease in expression with cohesin knockdown (translation). The bottom panel shows the heat map for all 959 genes that show significant increases in expression and all 1025 genes that show significant decreases in expression with Rad21 and Nipped-B RNAi knockdown. The significant GO categories for the affected genes are listed in Table S6, with the probe identities in each group. (1.01 MB TIF) [file pone.0006202.s011.tif]

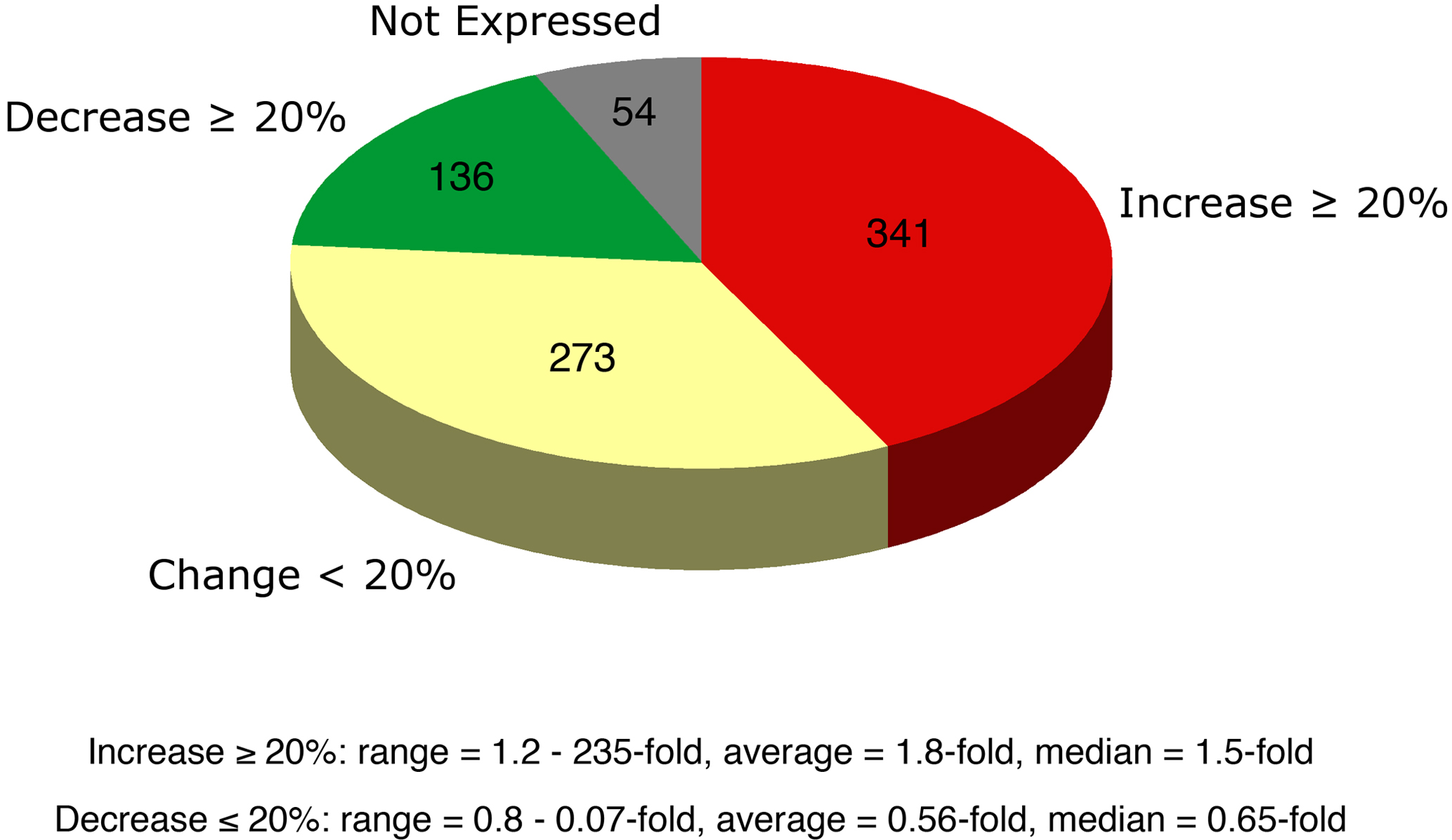

Supplement: Figure S4 — Effects of Rad21 on genes binding both cohesin and RNA polymerase II in BG3 cells. The 804 genes binding both PolII and cohesin whose expression was measured by the microarray are broken into four categories based on their response to Rad21 knockdown after six days, with the number of genes in each category indicated on the pie chart. (0.34 MB TIF) [file pone.0006202.s012.tif]
